# Impact of antibody-mediated rejection detected on one-year protocol biopsies on long-term kidney allograft survival

**DOI:** 10.3389/fmed.2026.1938644

**Published:** 2026-09-03

**Authors:** Galina Severova, Vlatko Karanfilovski, Lada Trajceska, Irena Rambabova-Bushljetik, Pavlina Dzekova-Vidimliski, Igor G. Nikolov, Nikola Gjorgjievski, Gordana Petrushevska, Slavica Kostadinova-Kunovska, Goce Spasovski, Ninoslav Ivanovski

**Affiliations:** 1 Faculty of Medicine, Saints Cyril and Methodius University of Skopje, Skopje, North Macedonia; 2 University Clinic od Nephrology, Skopje, North Macedonia; 3 Institute od Pathology, Skopje, North Macedonia

**Keywords:** antibody-mediated rejection, chronic, graft survival, kidney failure, kidney transplantation, protocol biopsy

## Abstract

**Introduction:**

Kidney transplantation undoubtedly remains the best way of treatment for end-stage chronic kidney disease. Enormous success has been achieved in improving short-term graft survival, but long-term graft survival has presented a great challenge, especially from an immunological perspective. This study aimed to evaluate the impact of antibody-mediated rejection (ABMR) detected on one-year protocol graft biopsies in kidney transplant recipients with stable graft function on 10-year death-censored graft survival and overall patient survival.

**Material and methods:**

Fifty adult kidney transplant recipients with stable graft function underwent a one-year protocol graft biopsy. According to the Banff 2013 classification, recipients were classified into ABMR and non-ABMR groups and followed until graft loss, death, or completion of the 10-year post-transplant follow-up. All of them were with low to moderate immunological risk, with no preformed Donor Specific Antibodies (DSA). The treatment consists of induction therapy, Anti-Thymocyte Globulin (ATG) or Basiliximab, and standard triple immunosuppressive therapy: steroids, Mycophenolate mofetil (MMF), and calcineurin inhibitors. Tissue samples were evaluated according to the BANFF 2013 classification by one pathologist. According to findings from graft biopsies, patients were divided into two groups: ABMR and non-ABMR. They were followed from the time of transplantation until graft loss, death, or completion of the 10-year post-transplant follow-up, whichever occurred first. We compared the two groups for demographic and other factors that could influence the immunological profile of the patient. Also, we evaluated the death-censored graft survival and overall survival of the patients.

**Results:**

Fifteen recipients (30%) had ABMR lesions on the one-year protocol biopsy, while 35 (70%) had no evidence of ABMR. Baseline demographic characteristics, immunological risk factors, immunosuppressive therapy, and graft function did not differ significantly between the groups. Recipients with ABMR had significantly inferior 10-year death-censored graft survival (log-rank *p* = 0.049). In univariate Cox regression, ABMR was associated with an increased risk of graft loss (HR 2.79, 95% CI 1.01–7.70; *p* = 0.047), while logistic regression demonstrated higher odds of graft failure (OR 3.86, 95% CI 1.07–13.94; *p* = 0.049). Overall patient survival did not differ significantly between the groups.

**Conclusion:**

In this cohort, ABMR detected on one-year protocol graft biopsies was associated with inferior 10-year death-censored graft survival in kidney transplant recipients with stable graft function. These findings suggest that one-year protocol biopsies may provide useful prognostic information for identifying recipients at increased risk of long-term graft loss. However, given the small sample size, limited number of graft failure events, and observational study design, these findings should be interpreted as an association rather than evidence of a definitive prognostic effect and require confirmation in larger, preferably multicenter cohorts.

## Introduction

Kidney transplantation undoubtedly remains the best treatment for end-stage chronic kidney disease. Advances in surgical techniques, immunosuppressive therapy, donor selection, and post-transplant monitoring have substantially improved short-term graft survival over the past decades. However, despite these achievements, long-term graft survival remains a major challenge, particularly from an immunological perspective (1, 2)..

The fundamental aspect of transplant immunology involves the human leukocyte antigen (HLA) system and its essential role in allograft recognition. Anti-HLA antibodies directed against donor antigens are referred to as donor-specific antibodies (DSA) and represent the principal mediators of antibody-mediated rejection (ABMR). Their interaction with the graft endothelium promotes complement activation, endothelial injury, microvascular inflammation, transplant glomerulopathy, and progressive chronic allograft dysfunction. These processes affect both glomerular and peritubular capillaries and may contribute to the development of interstitial fibrosis and tubular atrophy (IF/TA). Collectively, these lesions constitute the histopathological spectrum of ABMR. Together with T-cell-mediated rejection (TCMR) and other allograft lesions, they are classified according to the Banff criteria (3–5).

The development of *de novo* DSA reflects ongoing humoral alloimmunity and represents an important risk factor for chronic ABMR and subsequent graft loss. Memory B cells play a central role in this process because they retain immunological memory of previous exposure to foreign HLA antigens and can rapidly produce antibodies upon re-exposure. Consequently, post-transplant monitoring of anti-HLA antibodies has become an important component of long-term immunological surveillance. In addition, complement-dependent cytotoxicity (CDC) crossmatch testing and accurate pre-transplant detection of anti-HLA antibodies have significantly reduced the incidence of early acute ABMR (6).

The introduction of graft biopsies, particularly protocol biopsies in kidney transplant recipients with stable graft function, has provided direct insight into the histological status of the allograft and enabled correlation with immunological, biochemical, proteomic, and more recently molecular biomarkers such as donor-derived cell-free DNA. Protocol biopsies may reveal evidence of ongoing alloimmune injury despite stable clinical and biochemical graft function. The presence of ABMR lesions, either isolated or combined with other histological entities, suggests the existence of subclinical immune-mediated injury. Such findings are commonly referred to as subclinical rejection and may include both TCMR and ABMR lesions. Particularly valuable information is obtained from late protocol biopsies performed one year or more after transplantation. A considerable proportion of these biopsies are C4d-negative, suggesting that mechanisms other than classical complement activation may contribute to graft injury. The identification of such lesions is important because it may facilitate earlier therapeutic intervention and potentially improve long-term graft outcomes (3, 7, 8).

Regarding the timing of post-transplant protocol biopsies, no universally accepted protocol currently exists. Protocol biopsies are generally performed between three and twenty-four months after transplantation, while the one-year biopsy is considered particularly informative for the assessment of chronic lesions. The one-year time point was selected in our study because it represents an important transition between early post-transplant injury and the development of chronic allograft changes, when subclinical alloimmune injury may already be detectable despite stable graft function (9) In clinically stable recipients, the rationale for protocol biopsies remains debated because of procedural risks and the absence of overt clinical evidence of rejection. Nevertheless, accumulating evidence suggests that the information obtained from protocol biopsies may improve risk stratification, facilitate early therapeutic optimization, delay graft failure, and ultimately improve quality of life (2, 10–12).

Although protocol biopsies are increasingly incorporated into routine post-transplant surveillance, data regarding the long-term prognostic significance of ABMR detected in clinically stable recipients remain limited. In particular, studies evaluating the impact of ABMR identified on one-year protocol biopsies on 10-year graft survival are scarce. Therefore, the aim of this study was to evaluate the impact of ABMR detected on one-year graft biopsies on long-term graft survival and patient survival in kidney transplant recipients with stable graft function.

## Material and methods

### Study population

This observational single-center cohort study included adult kidney transplant recipients who underwent kidney transplantation between 2013 and 2015 and were subsequently followed at the University Clinic of Nephrology. One year after transplantation, all eligible recipients underwent a protocol graft biopsy. Patients were followed until graft loss, death, or completion of the 10-year post-transplant follow-up, whichever occurred first. The study was approved by the local Ethics Committee and conducted in accordance with the Declaration of Helsinki. Written informed consent was obtained from all participants before enrollment. Among the transplant recipients regularly followed at our center, 50 patients who underwent a protocol graft biopsy one year after kidney transplantation and fulfilled the study eligibility criteria were included in the analysis. No *a priori* sample size or power calculation was performed because this was an observational cohort study including all eligible recipients who underwent a one-year protocol biopsy during the study period. A total of 50 recipients met the predefined inclusion criteria and had adequate biopsy material and sufficient follow-up data for inclusion in the final analysis. Inclusion criteria were: age >18 years, low to moderate immunological risk, negative complement-dependent cytotoxicity (CDC) crossmatch, absence of preformed donor-specific antibodies (DSA) before transplantation, first kidney transplantation, stable graft function, and absence of infection or clinical evidence of rejection at the time of biopsy. Patients with inadequate biopsy specimens, defined as fewer than eight glomeruli, incomplete clinical data, or insufficient follow-up information were excluded from the study. One year after kidney transplantation, all recipients underwent a protocol graft biopsy. Biopsy specimens were evaluated according to the updated Banff 2013 criteria which was the classification applied at the time of the biopsies by the same renal pathologist. Based on the histopathological findings, recipients were classified into an ABMR group when ABMR was identified as a diagnostic category on the one-year protocol biopsy, either as an isolated diagnosis or as a component of a mixed histopathological diagnosis, and into a non-ABMR group when ABMR was not identified.

### Clinical and laboratory data

The following demographic and clinical variables were collected from medical records: recipient age, sex, primary kidney disease, history of pregnancy, blood transfusions, HLA mismatches, donor type, dialysis modality before transplantation, immunosuppressive therapy, and biochemical parameters. All recipients received induction therapy with either anti-thymocyte globulin (ATG) or basiliximab, followed by maintenance triple immunosuppressive therapy consisting of corticosteroids, mycophenolic acid, and a calcineurin inhibitor (cyclosporine A or tacrolimus). Therapeutic drug monitoring was routinely performed according to institutional protocols. Tacrolimus trough concentrations were generally maintained between 5 and 8 ng/mL, while cyclosporine levels were Aadjusted according to standard clinical recommendations. Anti-HLA antibodies were assessed at 3 and 12 months after transplantation and thereafter annually or whenever clinically indicated. DSA were identified using Luminex technology with single-antigen bead (SAB) assays. Graft function was assessed using serum creatinine concentration (µmol/L), estimated glomerular filtration rate (eGFR) calculated using the CKD-EPI equation (mL/min/1.73 m^2^), and 24-hour proteinuria (g/day).

### Follow-up and outcomes

Following the one-year protocol biopsy, recipients were followed until graft loss, death, or completion of the 10-year post-transplant follow-up, whichever occurred first. Graft failure was defined as return to chronic dialysis treatment or retransplantation. Death-censored graft survival was calculated by censoring patients at the time of death with a functioning graft. Management following the one-year protocol biopsy was individualized according to the histopathological findings and clinical status of the recipient. In recipients with subclinical ABMR and stable graft function without an increase in serum creatinine or significant proteinuria, maintenance immunosuppression was optimized or intensified, followed by close clinical and immunological surveillance. In recipients with ABMR accompanied by clinically evident graft dysfunction, more intensive anti-rejection treatment was administered according to institutional practice, including intravenous methylprednisolone pulse therapy and, in selected cases, plasmapheresis followed by rituximab. In selected cases, indication biopsies were performed during follow-up. The primary study endpoint was death-censored graft survival at 10 years following kidney transplantation. The secondary endpoint was overall patient survival.

### Statistical analysis

Continuous variables were expressed as mean ± standard deviation (SD) or median with interquartile range (IQR), depending on data distribution. Categorical variables were presented as frequencies and percentages. Comparisons between groups were performed using the Mann–Whitney U test for continuous variables and the chi-square test or Fisher's exact test for categorical variables, as appropriate. Kaplan–Meier survival analysis was used to evaluate death-censored graft survival and overall patient survival. Differences between survival curves were assessed using the log-rank test. A two-sided *p*-value <0.05 was considered statistically significant. Statistical analyses were performed using SPSS software.

## Results

The study cohort consisted of 50 kidney transplant recipients with a mean age of 37.4 ± 11.6 years. Thirty-six patients (72%) were male, and 47 (94%) received renal replacement therapy before transplantation. Baseline demographic and clinical characteristics of the cohort are presented in Table 1.

**Table 1:** Demographic characteristics of a study group *n* = 50.

1	Age (mean, SD)	37.39 ± 11.6
2	Gender (n/%) Male Female	36 (72%) 14 (28%)
3	Primary kidney disease	n (%)
	Arterial hypertension Diabetes mellitus Vesico-ureteral reflux APKD Chronic glomerulonephritis IgA nephropathy FSGS MPGN IPN Alport syndrome RPGN Membranous nephropathy SLE Unknown	1 (2%) 2 (4%) 2 (4%) 3 (6%) 11 (22%) 1 (2%) 4 (8%) 3 (6%) 2 (4%) 4 (8%) 4 (8%) 1 (2%) 1 (2%) 11 (22%)
4	Factors that can contribute to sensitizing	n (%)
	Pregnancy Blood transfusion HLA mismatch > 1	11 (78.6% of female recipients) 10 (20%) 10 (20%)
5	Dialysis before transplantation	n (%)
	Hemodialysis CAPD Preemptive	44 (88%) 3 (6%) 3 (6%)
6	Type of donor	n (%)
	Live Related Unrelated DCD	42 (84%) 40 (95.2%) 2 (4.8%) 8 (16%)

Adult Polycystic Kidney Disease (APKD), Focal Segmental Glomerulosclerosis (FSGS), Membranoproliferative Glomerulonephritis (MPGN), Interstitial-Pyelonephritis (IPN), Rapid Progressive Glomerulonephritis (RPGN), Systemic Lupus Erythematosus (SLE), Continuous Ambulatory Peritoneal Dialysis (CAPD).

At the time of protocol biopsy, all patients had clinically and biochemically stable values for graft function (mean serum creatinine 128.13 ± 36 µmol/L, proteinuria 0.36 ± 0.17 g/day; Glomerular Filtration Rate - GFR 72 ± 38 mL/min). *De novo* Anti-HLA antibodies were detected in 17 (34%) patients, and DSA was detected in 6 patients (3 in ABMR group and 3 in non-ABMR group). No complications of the graft biopsy were observed.

According to the Banff 2013 classification, normal histology was observed in 16 patients (32%), whereas 34 patients (68%) demonstrated at least one histopathological abnormality. The most common isolated lesions were borderline T-cell-mediated rejection, IF/TA, and category 6 lesions. Mixed histological findings were identified in 17 patients (34%), of whom 14 exhibited lesions compatible with ABMR. Among patients with mixed lesions, the most frequent combination was ABMR associated with IF/TA (41%), followed by ABMR combined with borderline rejection and IF/TA (29%). Pure ABMR was observed in only one recipient. (Tables 2, 3).

**Table 2:** Biopsy categorization according to the updated banff 2013 classification (*n* = 50).

Banff diagnostic category	N	%
Normal (Category 1)	16	32
Pure ABMR (Category 2)	1	2
Borderline T-cell rejection (Category 3)	5	10
T-cell rejection (Category 4)	1	2
IF/TA (Category 5)	5	10
Other (Category 6)	5	10
Mixed	17	34

**Table 3:** Analysis of the “mixed findings” category (*n* = 17).

Category	n	%
ABMR (cat 2) + IF/TA (cat 5)	7	41
ABMR (cat 2) + BL (cat 3)	1	5
ABMR (cаt 2) + BL (cat 3) + IF/TA (cat 5)	5	29
BL (cat 3) + IF/TA (cat 5)	2	11
ABMR (cat2) + TCMR (cat4)	1	5
TCMR (cat 4) + IF/TA (cat 5)	1	5

Borderline (BL), T- cell mediated rejection (TCMR).

Based on the histopathological findings of the one-year protocol biopsy, 15 recipients (30%) were classified into the ABMR group and 35 (70%) into the non-ABMR group. Among the 15 recipients in the ABMR group, the most frequent histopathological findings were tubular atrophy (13/15, 86.7%), interstitial fibrosis (12/15, 80.0%), and interstitial inflammation (11/15, 73.3%). Transplant glomerulopathy was present in 10/15 recipients (66.7%), while tubulitis was observed in 10/15 (66.7%) and intimal arteritis in 7/15 (46.7%). Regarding microvascular lesions, glomerulitis was present in 6/15 recipients (40.0%), peritubular capillaritis in 5/15 (33.3%), and microvascular inflammation in 5/15 (33.3%). C4d staining was positive in 4/15 recipients (26.7%). Notably, several DSA-negative recipients demonstrated prominent microvascular inflammation, with glomerulitis scores reaching g2 and peritubular capillaritis scores of ptc2–3, despite negative C4d staining. These groups were subsequently followed until graft loss, death, or completion of the 10-year post-transplant follow-up. No statistically significant differences were observed between the ABMR and non-ABMR groups regarding demographic characteristics, sensitization-related factors, donor type, HLA mismatch burden, induction therapy, maintenance immunosuppressive regimen, or baseline graft function, including serum creatinine, eGFR, and proteinuria (all *p* > 0.05), indicating that both groups were broadly comparable at baseline. (Table 4).

**Table 4:** Baseline characteristics according to ABMR status.

Variable	ABMR *n* = 15	Non-ABMR *n* = 35	*p* value
Sex Male Female	14 (93.3%) 1 (6.7%)	23 (65.7%); 12 (34.3%)	0.091
Age (years)	38.1 ± 13.1	36.4 ± 10.5	0.518
Dialysis Hemodialysis CAPD preemptive	14 (93.3%) 1 (6.7%) 0	30 (85.7%) 2 (5,7%) 3 (8.6%)	0.504
Pregnancies	1 (6.7%)	10 (28.6%)	0.139
Transfusions	2 (13.3%)	8 (22.9%)	0.702
Donor type Live related Live unrelated DCD	12 1 3	29 1 5	0.367
HLA mismatch	0.7 ± 0.6	0.6 ± 0.8	0.388
ATG	6 (40.0%)	11 (31.4%)	0.746
Basiliximab	9 (60.0%)	24 (68.6%)	0.746
Cyclosporine	9 (60.0%)	11 (31.4%)	0.114
Tacrolimus	6 (40.0%)	24 (68.6%)	0.114
Serum creatinine (*μ*mol/L)	115 (107.5–134.0)	110 (88.0–153.5)	0.511
24-hour proteinuria (g/day)	0.39 (0.12–0.56)	0.08 (0.07–0.50)	0.242
eGFR (mL/min/1.73m^2^)	77.25 (62.3–88.1)	70.63 (50.8–92.5)	0.751

The median post-transplant follow-up duration was 10 years (IQR 7–10 in the ABMR group and 8–10 in the non-ABMR group). During the follow-up, indication biopsies were performed in three recipients. BK polyomavirus nephropathy was diagnosed in one patient, whereas two patients developed chronic allograft nephropathy. Clinically apparent rejection occurred in two recipients. One patient was treated with pulse methylprednisolone, while the second developed ABO-incompatible rejection and was treated with five sessions of plasmapheresis followed by rituximab, resulting in stabilization of graft function in both cases. During follow-up, seven patients (14%) died. Causes of death included cardiovascular disease (*n* = 4), COVID-19-related complications (*n* = 2), and malignancy (*n* = 1). Overall, graft failure occurred in 16 recipients (32%); fifteen returned to maintenance hemodialysis and one underwent retransplantation. Graft failure occurred significantly more frequently among recipients with ABMR than among those without ABMR on the protocol biopsy (53.3% vs. 22.9%, *p* = 0.049). In contrast, overall mortality did not differ significantly between the groups (20.0% vs. 11.4%, *p* = 0.41). The duration of follow-up was comparable between the two groups (*p* = 0.930). (Table 5).

**Table 5:** ABMR vs. non-ABMR comparison of follow-up period, graft failure, and deaths. .

Variable	ABMR	Non-ABMR	*p* value
Post-transplant follow-up (years, median IQR)	10 [7–10]	10 [8–10]	0.930
graft failure n (%)	8 (53.3%)	8 (22.9%)	0.049
Death n(%)	3 (20.0%)	4 (11.4%)	0.415

### Kaplan–Meier death-censored graft survival

**Figure 1:**
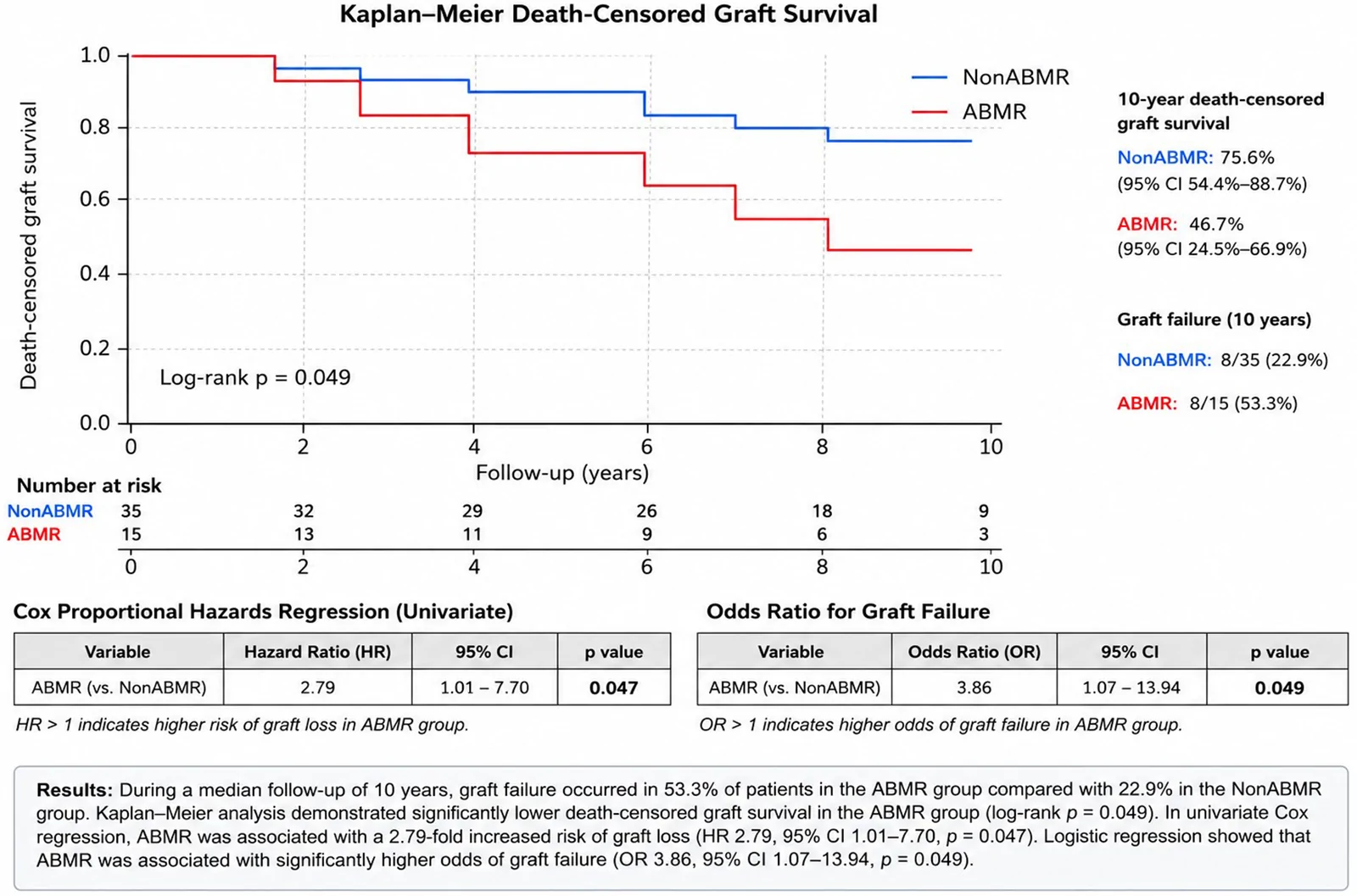
Death-censored graft survival according to findings on the one-year protocol biopsy (ABMR vs. non-ABMR): Kaplan–Meier, univariate Cox proportional hazards, and logistic regression analyses.

Kaplan–Meier analysis demonstrated significantly inferior death-censored graft survival in the ABMR group compared with the non-ABMR group (log-rank *χ*^2^ = 3.87, *p* = 0.049). In univariate Cox proportional hazards regression analysis, ABMR was associated with a significantly increased risk of graft loss (HR 2.79, 95% CI 1.01–7.70, *p* = 0.047). Logistic regression further demonstrated that ABMR was associated with higher odds of graft failure (OR 3.86, 95% CI 1.07–13.94, *p* = 0.049). (Figure 1).

### Overall patient survival

Kaplan–Meier analysis demonstrated 10-year overall patient survival of 77.9% in the ABMR group and 86.8% in the Non-ABMR group. Although overall survival appeared numerically lower in patients with ABMR, the difference between the survival curves was not statistically significant according to the log-rank test (*χ*^2^ = 0.509, *p* = 0.475).

## Discusion

In this observational single-center cohort study, we demonstrated that ABMR detected on one-year protocol graft biopsies was associated with inferior 10-year death-censored graft survival compared with recipients without ABMR. In addition, univariate Cox regression demonstrated a significantly increased hazard of graft loss among recipients with ABMR. These findings support the concept that antibody-mediated injury remains one of the principal determinants of long-term kidney allograft failure. Similar observations have been reported by Loupy et al. and Einecke et al., who identified microvascular injury associated with antibody-mediated mechanisms as a major contributor to late graft dysfunction and graft loss (6, 13, 14).

The fact that 34 (68%) of patients with clinically and biochemically stable graft function had histopathological changes suggests that the immune system can be “silently” active despite stable laboratory parameters. This represents one of the strongest justifications for performing protocol biopsies. Rush et al. and Nankivell et al. have shown that subclinical rejection is significantly associated with the further development of chronic damage and worse graft outcomes (15, 16).

In our study, pure ABMR was uncommon and identified in only one patient. In contrast, most ABMR cases occurred in combination with other Banff diagnostic categories, particularly IF/TA and borderline changes. These findings support the concept that chronic allograft injury is rarely the result of a single pathogenic mechanism. Rather, multiple immunological and non-immunological processes frequently coexist and contribute simultaneously to progressive graft damage. Similar observations have been reported in Banff consensus publications and studies by Haas et al. and Cosio et al., where mixed lesions were associated with ongoing chronic injury and reduced long-term graft survival (17–19).

Acute ABMR is most often directly associated with the presence of donor-specific antibodies (DSA), especially in the early posttransplant period. Interestingly, DSA were not detected in all recipients classified in the ABMR group. Several DSA-negative recipients demonstrated prominent microvascular inflammation, with glomerulitis scores reaching g2 and peritubular capillaritis scores of ptc2–3, despite negative C4d staining. These findings highlight the complexity of microvascular injury in kidney allografts and the limitations of relying on circulating DSA alone to characterize the underlying immunological process. Although non-HLA antibodies, transient or low-level antibodies, and other mechanisms have been proposed as potential contributors to DSA-negative microvascular inflammation, these mechanisms could not be assessed in our cohort. Therefore, these findings should be interpreted cautiously and should not be considered evidence that DSA-negative cases necessarily represent antibody-mediated rejection. These patients actually belong to the entity of subclinical or silent rejection, which is most often associated with reduced immunosuppression due to infections or malignancies, as well as non-adherence. Wiebe et al. showed that *de novo* DSA is a strong predictor of chronic ABMR and graft loss (20). In addition, Zhang et al. and Dragun et al. suggest that non-HLA antibodies may play an important role in C4d-negative ABMR (21, 22).

The main role of antibodies is the interaction with the endothelium of glomerular and peritubular capillaries, which leads to microvascular inflammation and the development of transplant glomerulopathy. Today, the microvascular inflammation score (g + ptc) is considered one of the most important prognostic parameters for the risk of graft loss. Loupy et al. showed that high values ​​of the microvascular inflammation score significantly correlate with poorer long-term survival (23).

Since ABMR often occurs in association with IF/TA, it is possible that microvascular damage may lead to changes in local interstitial perfusion and promote fibrosis and tubular atrophy. Borderline changes, although less common, may indicate concurrent T-cell-mediated immune activation. Haas et al. have shown that a proportion of ABMR cases may be C4d-negative and without detectable anti-HLA antibodies, further highlighting the complexity of the pathogenesis (19).

When comparing the ABMR and non-ABMR groups, variables such as recipient age, sex, pregnancies, previous blood transfusions, HLA mismatch, donor type, induction therapy, maintenance immunosuppressive regimen, and baseline graft function, including serum creatinine, eGFR, and proteinuria, did not differ significantly between the groups. These findings indicate that both groups were broadly comparable at baseline, suggesting that the observed difference in long-term graft survival is more likely attributable to the presence of ABMR detected on the one-year protocol biopsy rather than to differences in baseline clinical or immunological characteristics. Although HLA mismatch and previous sensitization are traditionally considered important risk factors for alloimmune injury, Opelz and Döhler demonstrated that their impact may vary according to the immunosuppressive regimen and duration of follow-up (24).

During follow-up, one patient developed BK polyomavirus nephropathy with progressive graft dysfunction despite reduction of immunosuppressive therapy. BK polyomavirus nephropathy remains one of the most important infectious complications after kidney transplantation and represents a well-recognized cause of chronic allograft dysfunction and graft loss, as previously reported by Hirsch and Randhawa (25).

A total of 14% of recipients died during follow-up, most commonly from cardiovascular disease and COVID-19-related complications. Although overall patient survival appeared numerically lower in the ABMR group, Kaplan–Meier analysis demonstrated no statistically significant difference between the two groups. Cardiovascular disease remains the leading cause of death after kidney transplantation, while the COVID-19 pandemic further increased mortality among immunosuppressed recipients, consistent with previous reports by Kasiske et al., Meier-Kriesche et al., Caillard et al., and Cravedi et al. (26–29)

Our findings further emphasize the value of one-year protocol graft biopsies as the most accurate method for assessing the true histological status of the kidney allograft, even in recipients with stable clinical and biochemical graft function. In our cohort, protocol biopsies identified ABMR lesions associated with inferior long-term graft survival despite the absence of clinical signs of rejection at the time of biopsy. Although biopsy-related complications have been reported in the literature, no major or minor complications were observed in our study. Schwarz et al. similarly demonstrated that protocol biopsies are a safe procedure with a very low complication rate (30).

The main strengths of our study include the use of one-year protocol biopsies in clinically stable kidney transplant recipients, longitudinal assessment of donor-specific antibodies, consistent histopathological evaluation by the same renal pathologist, and long-term follow-up extending to 10 years. The use of death-censored graft survival as the primary endpoint also allowed us to assess the association between histopathological findings at one year and subsequent graft failure independently of death with a functioning graft. This study has several limitations, including the relatively small sample size, the single-center design, and the lack of additional modern biomarkers such as non-HLA antibodies, donor-derived cell-free DNA, proteomics, and molecular microscopy. Post-biopsy management was individualized according to clinical status and histopathological findings rather than standardized, and differences in subsequent immunosuppressive management may have influenced long-term graft outcomes. Furthermore, because of the limited number of graft failure events, multivariable Cox regression analysis could not be performed. The absence of contemporary molecular biomarkers represents an additional limitation, as molecular diagnostics and donor-derived cell-free DNA may provide complementary information for the characterization and early detection of allograft injury (31, 32).

## Conclusion

In this study, ABMR detected on one-year protocol graft biopsies was associated with inferior long-term death-censored graft survival, even in kidney transplant recipients with stable clinical and biochemical graft function. These findings suggest that one-year protocol biopsies may provide useful prognostic information for identifying recipients at increased risk of chronic graft injury and graft loss. However, the findings should be interpreted cautiously given the small sample size, limited number of graft failure events, observational design, and predominance of mixed rather than isolated ABMR lesions. Larger multicenter studies are needed to confirm the prognostic significance of ABMR detected on protocol biopsy.

## Data Availability

The raw data supporting the conclusions of this article will be made available by the authors, without undue reservation.
